# Vaccination Coverage by Age 24 Months Among Children Born During 2018–2019 — National Immunization Survey–Child, United States, 2019–2021

**DOI:** 10.15585/mmwr.mm7202a3

**Published:** 2023-01-13

**Authors:** Holly A. Hill, Michael Chen, Laurie D. Elam-Evans, David Yankey, James A. Singleton

**Affiliations:** 1 Immunization Services Division, National Center for Immunization and Respiratory Diseases, CDC.

Millions of young children are vaccinated safely in the United States each year against a variety of potentially dangerous infectious diseases (*1*). The Advisory Committee on Immunization Practices (ACIP) recommends routine vaccination against 14 diseases during the first 24 months of life* (*2*). This report describes vaccination coverage by age 24 months using data from the National Immunization Survey–Child (NIS-Child).^†^ Compared with coverage among children born during 2016–2017, coverage among children born during 2018–2019 increased for a majority of recommended vaccines. Coverage was >90% for ≥3 doses of poliovirus vaccine (93.4%), ≥3 doses of hepatitis B vaccine (HepB) (92.7%), ≥1 dose of measles, mumps, and rubella vaccine (MMR) (91.6%), and ≥1 dose of varicella vaccine (VAR) (91.1%); coverage was lowest for ≥2 doses of hepatitis A vaccine (HepA) (47.3%). Vaccination coverage overall was similar or higher among children reaching age 24 months during March 2020 or later (during the COVID-19 pandemic) than among those reaching age 24 months before March 2020 (prepandemic); however, coverage with the combined 7-vaccine series^§^ among children living below the federal poverty level or in rural areas decreased by 4–5 percentage points during the pandemic (*3*). Among children born during 2018–2019, coverage disparities were observed by race and ethnicity, poverty status, health insurance status, and Metropolitan Statistical Area (MSA) residence. Coverage was typically higher among privately insured children than among children with other insurance or no insurance. Persistent disparities by health insurance status indicate the need to improve access to vaccines through the Vaccines for Children (VFC) program.^¶^ Providers should review children’s histories and recommend needed vaccinations during every clinical encounter and address parental hesitancy to help reduce disparities and ensure that all children are protected from vaccine-preventable diseases.

NIS-Child is a random-digit–dialed survey of households that includes children aged 19–35 months. Parents or guardians complete a telephone survey,** and consent to contact the child’s vaccination providers is requested. With parental or guardian consent, identified providers are mailed a questionnaire to obtain vaccination information, which is synthesized to create the child’s comprehensive vaccination history. Children born during 2018–2019 were identified from data collected during 2019–2021, resulting in 29,598 children with adequate provider data^††^ for analysis. The 2021 household response rate^§§^ was 22.9%, and adequate provider data were obtained from 51.5% of households with completed interviews. Vaccination coverage by age 24 months was estimated using Kaplan-Meier techniques, except for the birth dose of HepB^¶¶^ and rotavirus vaccine.*** Coverage with ≥2 doses of HepA was also estimated by age 35 months (the maximum age available).^†††^ Significance of coverage differences was assessed using z-tests; p-values <0.05 were considered statistically significant. Analyses used weighted data and were performed using SAS (version 9.4; SAS Institute) and SUDAAN (version 11; RTI International). This activity was reviewed by CDC and was conducted consistent with applicable federal law and CDC policy.^§§§^

## National Vaccination Coverage

Among children born during 2018–2019, vaccination coverage by age 24 months increased compared with that among children born during 2016–2017 for a majority of vaccines (Table 1). Coverage was >90% for ≥3 doses of poliovirus vaccine (93.4%), ≥1 dose of MMR (91.6%), ≥3 doses of HepB (92.7), and ≥1 dose of VAR (91.1%). The only vaccines for which coverage was <70% were ≥2 doses of HepA (47.3%) and ≥2 doses of influenza vaccine (63.9%). The proportion of children who received no vaccinations by age 24 months decreased from 1.3% among those born during 2016–2017 to 0.9% among those born during 2018–2019. Coverage by birth year during 2011–2019 was stable for a majority of vaccines, with increases during recent years for the HepB birth dose, rotavirus vaccine, ≥2 influenza vaccine doses, and ≥2 HepA doses by age 35 months (Figure).

**TABLE 1:** Estimated vaccination coverage by age 24 months,* among children born during 2016–2017 and during 2018–2019 for selected vaccines and doses — National Immunization Survey–Child, United States, 2017–2021

Vaccine and dose	% (95% CI)		
	Birth year^†^		Percentage point difference
	2016–2017	2018–2019	(2016–2017 to 2018–2019)
**DTaP^§^**			
≥3 doses	93.2 (92.6 to 93.7)	94.2 (93.6 to 94.8)	1.0 (0.2 to 1.9)^¶^
≥4 doses	80.6 (79.7 to 81.6)	81.9 (80.9 to 82.8)	1.3 (−0.1 to 2.6)
**Poliovirus (≥3 doses)**	92.0 (91.4 to 92.6)	93.4 (92.8 to 94.0)	1.4 (0.5 to 2.2)^¶^
MMR (≥1 dose)**	90.6 (89.9 to 91.3)	91.6 (90.9 to 92.3)	1.1 (0.1 to 2.0)^¶^
**Hib** ^††^			
Primary series	92.4 (91.7 to 93.0)	93.6 (93.0 to 94.1)	1.2 (0.3 to 2.1)^¶^
Full series	79.6 (78.6 to 80.6)	80.0 (79.0 to 81.0)	0.4 (−1.0 to 1.8)
**HepB**			
Birth dose^§§^	76.4 (75.4 to 77.4)	79.8 (78.8 to 80.8)	3.4 (2.0 to 4.8)^¶^
≥3 doses	91.2 (90.6 to 91.9)	92.7 (92.0 to 93.3)	1.4 (0.5 to 2.3)^¶^
VAR (≥1 dose)**	90.1 (89.4 to 90.8)	91.1 (90.3 to 91.8)	1.0 (0 to 2.0)
**PCV**			
≥3 doses	91.7 (91.0 to 92.3)	93.3 (92.7 to 93.9)	1.7 (0.8 to 2.5)^¶^
≥4 doses	81.2 (80.2 to 82.1)	83.5 (82.6 to 84.4)	2.3 (1.0 to 3.7)^¶^
**HepA**			
≥1 dose	85.6 (84.8 to 86.4)	88.3 (87.5 to 89.1)	2.7 (1.5 to 3.8)^¶^
≥2 doses^¶¶^	45.2 (44.0 to 46.4)	47.3 (46.0 to 48.5)	2.1 (0.3 to 3.8)^¶^
≥2 doses (by age 35 mos)^¶¶^	76.8 (75.6 to 78.1)	79.6 (78.0 to 81.0)	2.7 (0.8 to 4.7)^¶^
**Rotavirus** (by age 8 mos)***	74.6 (73.5 to 75.6)	77.1 (76.1 to 78.2)	2.6 (1.1 to 4.1)^¶^
**Influenza (≥2 doses)^†††^**	57.5 (56.3 to 58.6)	63.9 (62.7 to 65.0)	6.4 (4.8 to 8.0)^¶^
**Combined 7-vaccine series^§§§^**	69.8 (68.6 to 70.9)	70.1 (68.9 to 71.2)	0.3 (−1.3 to 1.9)
**No vaccinations^¶¶¶^**	1.3 (1.1 to 1.5)	0.9 (0.7 to 1.1)	−0.4 (−0.7 to −0.1)^¶^

**Abbreviations:** DTaP = diphtheria, tetanus toxoids, and acellular pertussis vaccine; HepA = hepatitis A vaccine; HepB = hepatitis B vaccine; Hib = *Haemophilus influenzae* type b conjugate vaccine; MMR = measles, mumps, and rubella vaccine; PCV = pneumococcal conjugate vaccine; RV5 = pentavalent rotavirus vaccine; VAR = varicella vaccine.

* Includes vaccinations received by age 24 months (before the day the child turns age 24 months), except for the HepB birth dose, rotavirus vaccination, and ≥2 HepA doses by age 35 months. For all vaccines except the HepB birth dose and rotavirus vaccination, the Kaplan-Meier method was used to estimate vaccination coverage to account for children whose vaccination history was ascertained before age 24 months (age 35 months for ≥2 HepA doses).

^†^ Data for the 2016 birth year are from survey years 2017, 2018, and 2019; data for the 2017 birth year are from survey years 2018, 2019, and 2020; data for the 2018 birth year are from survey years 2019, 2020, and 2021; data for the 2019 birth year are considered preliminary and are from survey years 2020 and 2021 (data from survey year 2022 are not yet available).

^§^ Includes children who might have received diphtheria and tetanus toxoids vaccine or diphtheria, tetanus toxoids, and pertussis vaccine. Healthy People 2030 target for ≥4 doses of DTaP is 90%. https://health.gov/healthypeople/objectives-and-data/browse-objectives/vaccination

^¶^ Statistically significantly different from 0 at p<0.05.

** Includes children who might have received measles, mumps, rubella, and varicella combination vaccine. Healthy People 2030 target for ≥1 dose of MMR is 90.8%. https://health.gov/healthypeople/objectives-and-data/browse-objectives/vaccination

^††^ Hib primary series: receipt of ≥2 or ≥3 doses, depending on product type received; full series: primary series and booster dose, which includes receipt of ≥3 or ≥4 doses, depending on product type received.

^§§^ One dose HepB administered from birth through age 3 days.

^¶¶^ Before 2020, first dose of HepA was recommended at age 12–23 months, with second dose administered 6–18 months after the first, depending upon the product type received. During 2020, recommendation was revised to 2 doses between ages 12 and 23 months, ≥6 months apart. Because children in this analysis were vaccinated under both recommendations, coverage estimates for both <24 months and <35 months are provided.

*** Includes ≥2 doses of Rotarix monovalent rotavirus vaccine or ≥3 doses of RotaTeq RV5. If any dose in the series is either RV5 or unknown, the default is a 3-dose series. The maximum age for the final rotavirus dose is age 8 months, 0 days.

^†††^ Doses must be ≥24 days apart (4 weeks with a 4-day grace period); doses could have been received during two influenza seasons.

^§§§^ The combined 7-vaccine series (4:3:1:3*:3:1:4) includes ≥4 doses of DTaP, ≥3 doses of poliovirus vaccine, ≥1 dose of measles-containing vaccine, the full Hib series (≥3 or ≥4 doses, depending on product type), ≥3 doses of HepB, ≥1 dose of VAR, and ≥4 doses of PCV.

^¶¶¶^ Healthy People 2030 target for children who get zero recommended vaccines by age 2 years is 1.3%. https://health.gov/healthypeople/objectives-and-data/browse-objectives/vaccination

**FIGURE:**
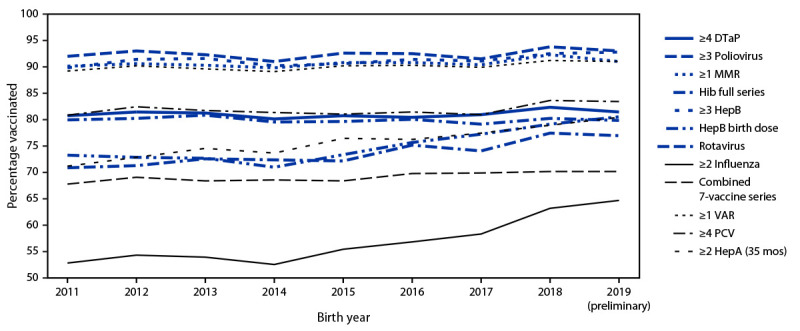
Estimated vaccination coverage with selected individual vaccines*^,†,§,¶,^**^,^^††^ and a combined vaccine series^§§^ by age 24 months,^¶¶^ by birth year 2011–2019*** — National Immunization Survey–Child, United States, 2012–2021 **Abbreviations:** DTaP = diphtheria, tetanus toxoids, and acellular pertussis vaccine; HepA = hepatitis A vaccine; HepB = hepatitis B vaccine; Hib = *Haemophilus influenzae* type b conjugate vaccine; MMR = measles, mumps, and rubella vaccine; PCV = pneumococcal conjugate vaccine; VAR = varicella vaccine. * Four or more DTaP includes children who might have received diphtheria and tetanus toxoids vaccine or diphtheria, tetanus toxoids, and pertussis vaccine. ^†^ One or more MMR includes children who might have received measles, mumps, rubella, and varicella combination vaccine. ^§^ Hib full series: primary series and booster dose, which includes receipt of ≥3 or ≥4 doses, depending on product type received. ^¶^ HepB birth dose = 1 dose HepB administered from birth through age 3 days. ** Rotavirus vaccination includes ≥2 doses of Rotarix monovalent rotavirus vaccine, or ≥3 doses of RotaTeq pentavalent rotavirus vaccine. The maximum age for the final rotavirus dose is 8 months, 0 days. ^††^ Influenza vaccine doses must be administered ≥24 days apart (4 weeks with a 4-day grace period); doses could have been received during two influenza seasons. ^§§^ The combined 7-vaccine series (4:3:1:3*:3:1:4) includes ≥4 doses of DTaP, ≥3 doses of poliovirus vaccine, ≥1 dose of measles-containing vaccine, the full series of Hib (≥3 or ≥4 doses, depending on product type), ≥3 doses of HepB, ≥1 dose of VAR, and ≥4 doses of PCV. ^¶¶^ Includes vaccinations received before age 24 months, except for the HepB birth dose, rotavirus vaccination, and ≥2 HepA doses by age 35 months. For all vaccines except the HepB birth dose and rotavirus vaccination, the Kaplan-Meier method was used to estimate vaccination coverage to account for children whose vaccination history was ascertained before age 24 months (35 months for ≥2 HepA doses). *** Children born in 2011 are included in survey years 2012, 2013, and 2014; children born in 2012 are included in survey years 2013, 2014, and 2015; children born in 2013 are included in survey years 2014, 2015, and 2016; children born in 2014 are included in survey years 2015, 2016, and 2017; children born during 2015 are included in survey years 2016, 2017, and 2018; children born in 2016 are included in survey years 2017, 2018, and 2019; children born in 2017 are included in survey years 2018, 2019 and 2020; children born in 2018 are included in survey years 2019 and 2020, and 2021; data for children born during 2019 are considered preliminary and are included in survey years 2020 and 2021 (data from survey year 2022 are not yet available).

## Vaccination by Selected Sociodemographic Characteristics and Geographic Locations

Among children born during 2018–2019, coverage among those who were uninsured and those insured by Medicaid or other insurance^¶¶¶^ was lower than that among privately insured children for all vaccines except the HepB birth dose, which was lower among uninsured children only (Table 2). The proportion of children who were unvaccinated by age 24 months was eight times higher for uninsured compared with privately insured children. Compared with non-Hispanic White children, coverage with a majority of vaccines was lower among non-Hispanic Black or African American (Black) children, and coverage with ≥1 MMR dose, ≥1 VAR dose, rotavirus vaccine, ≥2 influenza vaccine doses, and the 7-vaccine series was lower among Hispanic or Latino (Hispanic) children (Supplementary Table 1, https://stacks.cdc.gov/view/cdc/123206). Coverage was lower among children living below the poverty level than among those living at or above the poverty level for all vaccines except the HepB birth dose (Supplementary Table 2, https://stacks.cdc.gov/view/cdc/123207). Coverage with all vaccines except for the HepB birth dose was lower among children living in a non-MSA**** compared with those in an MSA principal city. Vaccination coverage varied widely by jurisdiction (Supplementary Table 3, https://stacks.cdc.gov/view/cdc/123208), especially coverage with ≥2 influenza vaccine doses, which ranged from 39.7% (Alabama) to 84.0% (Rhode Island).

**TABLE 2:** Estimated vaccination coverage by age 24 months* among children born during 2018–2019,^†^ by selected vaccines and doses and health insurance status^§^ — National Immunization Survey–Child, United States, 2019–2021

Vaccine and dose	Health insurance status, % (95% CI)			
	Private only (Ref) (n = 16,629)	Any Medicaid (n = 10,200)	Other insurance (n = 2,168)	Uninsured (n = 601)
**DTaP^¶^**				
≥3 doses	96.9 (96.4–97.4)	92.3 (91.2–93.2)**	92.7 (90.3–94.7)**	85.5 (80.6–89.7)**
≥4 doses	88.6 (87.6–89.6)	77.1 (75.4–78.8)**	78.9 (75.3–82.3)**	57.0 (49.1–65.0)**
**Poliovirus (≥3 doses)**	96.1 (95.5–96.6)	91.4 (90.3–92.5)**	91.9 (89.4–94.1)**	84.8 (79.7–89.1)**
**MMR (≥1 dose)^††^**	95.1 (94.4–95.7)	89.2 (87.9–90.4)**	90.0 (87.2–92.5)**	79.7 (73.0–85.6)**
**Hib** ^§§^				
Primary series	96.7 (96.1–97.1)	91.3 (90.2–92.4)**	92.4 (90.0–94.4)**	83.4 (77.9–88.2)**
Full series	86.2 (85.0–87.3)	75.6 (74.0–77.3)**	76.8 (73.2–80.3)**	57.8 (50.0–65.9)**
**HepB**				
Birth dose^¶¶^	80.6 (79.1–81.9)	79.8 (78.2–81.4)	77.4 (74.1–80.5)	69.9 (61.7–77.1)**
≥3 doses	94.6 (93.9–95.3)	91.4 (90.3–92.4)**	91.2 (88.7–93.3)**	84.6 (79.4–89.0)**
**VAR (≥1 dose)^††^**	94.3 (93.6–95.0)	89.1 (87.7–90.3)**	88.8 (85.9–91.4)**	76.8 (69.9–83.1)**
**PCV**				
≥3 doses	96.2 (95.6–96.8)	91.3 (90.2–92.4)**	91.1 (88.6–93.3)**	83.9 (78.6–88.5)**
≥4 doses	90.0 (89.0–91.0)	78.8 (77.2–80.3)**	80.6 (77.3–83.7)**	62.3 (54.9–69.7)**
**HepA**				
≥1 dose	91.3 (90.4–92.2)	86.3 (84.9–87.7)**	87.0 (84.4–89.4)**	73.2 (66.1–79.8)**
≥2 doses***	49.6 (47.9–51.3)	46.3 (44.3–48.3)**	45.4 (41.2–49.7)	27.9 (21.5–35.8)**
≥2 doses (by age 35 mos)***	84.9 (83.0–86.7)	76.2 (73.6–78.7)**	79.1 (73.9–83.8)**	43.4 (34.9–52.9)**
**Rotavirus (by age 8 mos)^†††^**	85.1 (83.9–86.2)	71.1 (69.3–72.8)**	72.0 (67.8–75.8)**	63.8 (56.4–70.7)**
**Influenza (≥2 doses)^§§§^**	77.1 (75.7–78.4)	52.6 (50.6–54.5)**	63.5 (59.4–67.6)**	40.1 (33.0–48.0)**
**Combined 7-vaccine series^¶¶¶^**	78.0 (76.6–79.4)	64.2 (62.3–66.1)**	67.4 (63.4–71.2)**	45.2 (37.8–53.3)**
**No vaccinations**	0.7 (0.5–0.9)	0.9 (0.6–1.3)	1.0 (0.6–1.7)	6.0 (3.2–10.0)**

**Abbreviations:** DTaP = diphtheria, tetanus toxoids, and acellular pertussis vaccine; HepA = hepatitis A vaccine; HepB = hepatitis B vaccine; Hib = *Haemophilus influenzae* type b conjugate vaccine; MMR = measles, mumps, and rubella vaccine; PCV = pneumococcal conjugate vaccine; Ref = referent group; VAR = varicella vaccine.

* Includes vaccinations received by age 24 months (before the day the child turns 24 months), except for the HepB birth dose, rotavirus vaccination, and ≥2 HepA doses by 35 months. For all vaccines except the HepB birth dose and rotavirus vaccination, the Kaplan-Meier method was used to estimate vaccination coverage to account for children whose vaccination history was ascertained before age 24 months (35 months for ≥2 HepA doses).

^†^ Data for the 2018 birth year are from survey years 2019, 2020, and 2021; data for the 2019 birth year are considered preliminary and are from survey years 2020 and 2021 (data from survey year 2022 are not yet available).

^§^ Children’s health insurance status was reported by parent or guardian. “Other insurance” includes the Children’s Health Insurance Program, military insurance, coverage via the Indian Health Service, and any other type of health insurance not mentioned elsewhere.

^¶^ Includes children who might have received diphtheria and tetanus toxoids vaccine or diphtheria, tetanus toxoids, and pertussis vaccine.

** Statistically significant (p<0.05) difference compared with Ref.

^††^ Includes children who might have received MMR and VAR combination vaccine.

^§§^ Hib primary series: receipt of ≥2 or ≥3 doses, depending on vaccine product type received; full series: primary series and booster dose, which includes receipt of ≥3 or ≥4 doses, depending on vaccine product type received.

^¶¶^ One dose HepB administered from birth through age 3 days.

*** Before 2020, first dose of HepA recommended at age 12–23 months, with second dose administered 6–18 months after the first, depending upon the vaccine product type received. During 2020, recommendation revised to 2 doses between ages 12 and 23 months, ≥6 months apart. Because children in this analysis were vaccinated under both recommendations, coverage estimates for both <24 months and <35 months are provided.

^†††^ Includes ≥2 doses of Rotarix monovalent rotavirus vaccine, or ≥3 doses of RotaTeq pentavalent rotavirus vaccine. If any dose in the series is either RotaTeq or unknown, the default is a 3-dose series. The maximum age for the final rotavirus dose is 8 months, 0 days.

^§§§^ Doses must be ≥24 days apart (4 weeks with a 4-day grace period); doses could have been received during two influenza seasons.

^¶¶¶^ The combined 7-vaccine series (4:3:1:3*:3:1:4) includes ≥4 doses of DTaP, ≥3 doses of poliovirus vaccine, ≥1 dose of measles-containing vaccine, the full series of Hib (≥3 or ≥4 doses, depending on product type), ≥3 doses of HepB, ≥1 dose of VAR, and ≥4 doses of PCV.

## Discussion

U.S. coverage with most recommended childhood vaccines has remained high and stable for many years. Increases in coverage by age 24 months were observed for most vaccines when comparing children born during 2018–2019 with those born during 2016–2017. Approximately 70% of children born in recent years (2016–2019) were up to date with the 7-vaccine series by age 24 months, with coverage >70% for all other vaccines except for ≥2 influenza vaccine doses and ≥2 doses of HepA. The proportion of children completely unvaccinated by age 24 months was 0.9% for children born during 2018–2019, meeting the Healthy People 2030^††††^ objective of <1.3%.

This report did not identify any overall decline in vaccination coverage associated with the COVID-19 pandemic among all children. The youngest children were born in 2019. These children reached age 12 months in 2020 and 24 months in 2021; therefore, many of these children had vaccine doses recommended after the pandemic was declared in March 2020. In a more detailed analysis, coverage with the combined 7-vaccine series by age 24 months decreased 4–5 percentage points among children living below the federal poverty level or in rural areas (*3*). In addition, MMR coverage was 10 percentage points lower for children reaching age 13 months during April–May 2020 compared with those reaching age 13 months before and after this time frame, but coverage reached prepandemic levels by age 19 months (*3*). Similar decreases in coverage were observed in other data sources (*4*). The 2022 NIS-Child will include more children born shortly before or during the pandemic, providing a more complete assessment of trends in vaccination coverage during the pandemic.

Vaccination coverage declined for children living below the federal poverty level or in rural areas during the pandemic, and substantial variation in coverage by sociodemographic characteristics persists. As observed elsewhere (*4*), estimated coverage was highest among Asian children and lowest among Black children. Lower coverage was found among children living below the federal poverty level, without private health insurance, and in rural (non-MSA) areas.

If equity is to be achieved in the national childhood vaccination program, a number of obstacles must be overcome. Parents and other caregivers must have the willingness and the means to get children vaccinated. A recent report estimated that 6.5%–31.3% of nonvaccination among children could be attributed to parental hesitancy, depending upon the vaccine (*5*). CDC has developed a Vaccinate with Confidence strategy for identifying activities designed to bolster vaccine confidence and prevent outbreaks of vaccine-preventable diseases (*6*). Several additional evidence-based approaches to increasing vaccination coverage include strong health care provider recommendations, advocating for vaccines at every health care encounter, use of reminder and recall notices and standing orders, and the presence of state and local immunization information systems to provide consolidated immunization histories (*7*).

Logistical and financial barriers also must be addressed. The VFC program covers the cost of all recommended vaccines for eligible children; it is imperative that this program retain an adequate supply of participating vaccination providers and that families in need are aware of how to access it. Establishment of alternative vaccination settings such as pharmacies, emergency departments, hospitals, and outpatient subspecialty clinics might help address accessibility issues for underserved communities (*8*).

The findings in this report are subject to at least three limitations. First, the possibility of selection bias exists because of the low household interview response rate (ranging from 21%–26% during survey years 2017–2021) and the availability of adequate provider data for 49%–54% of those who completed interviews in survey years 2017–2021. Second, although the data were weighted to account for nonresponse and households without telephones, some bias could remain. Finally, coverage estimates could be incorrect if some vaccination providers did not return questionnaires or if administered vaccines were not documented accurately. Total survey error (*9*) for the 2021 survey year data was assessed and demonstrated that coverage was underestimated by 3.1 percentage points for ≥1 dose of MMR, 4.4 percentage points for the HepB birth dose, and 8.7 percentage points for the combined -vaccine series. An analysis of change in bias of vaccination coverage estimates from 2020 to 2021 determined that a meaningful change was unlikely.^§§§§^

At the national level, coverage with most routine childhood vaccines is high; however, this high coverage is not distributed uniformly: coverage is lower among Black and Hispanic children, those of lower socioeconomic status, and those living in rural areas. Recent measles outbreaks^¶¶¶¶^ and the diagnosis of a case of polio (*10*) serve as reminders that pockets of susceptibility can and do exist, even in a largely well-vaccinated society. Parents and providers must remain vigilant to ensure that all children are up to date with their routine vaccinations to protect them from vaccine-preventable diseases.

**Summary**

**What is already known about this topic?**

The Advisory Committee on Immunization Practices recommends routine vaccination against 14 diseases during the first 24 months of life.

**What is added by this report?**

Vaccination coverage among young children has remained high and stable for most vaccines, although disparities persist. The National Immunization Survey–Child identified no decline overall in routine vaccination coverage associated with the COVID-19 pandemic among children born during 2018–2019, although declines were observed among children living below the federal poverty level and in rural areas.

**What are the implications for public health practice?**

Additional efforts, such as providers reviewing children’s immunization histories during every clinical encounter, recommending needed vaccinations, and addressing parental hesitancy, are warranted to reduce disparities so that all children can be protected from vaccine-preventable diseases.

## Footnotes

* Vaccination against COVID-19 was recommended for children aged 6 months–4 years in June 2022 (https://www.cdc.gov/vaccines/acip/recs/grade/covid-19-moderna-pfizer-children-vaccine-etr.html). Children in this report were either aged >4 years during June 2022 (some were eligible for COVID-19 vaccine at age ≥5 years, but vaccine history was not ascertained past age 35 months), or data on vaccine histories were collected before 2022.

† Estimates for U.S. Department of Health and Human Services regions, states, selected local areas, and the territories of Guam and Puerto Rico can be found online (https://www.cdc.gov/vaccines/imz-managers/coverage/childvaxview/data-reports/index.html). Certain local areas that receive federal Section 317 immunization funds are sampled separately and included in the NIS-Child sample every year (Chicago, Illinois; New York, New York; Philadelphia County, Pennsylvania; Bexar County, Texas; and Houston, Texas). National estimates in this report exclude territories.

§ The combined 7-vaccine series (4:3:1:3*:3:1:4) includes ≥4 doses of diphtheria and tetanus toxoids and acellular pertussis vaccine; ≥3 doses of poliovirus vaccine; ≥1 dose of measles-containing vaccine; ≥3 or ≥4 doses (depending upon product type) of *Haemophilus influenzae* type b conjugate vaccine; ≥3 doses of hepatitis B vaccine; ≥1 dose of varicella vaccine; and ≥4 doses of pneumococcal conjugate vaccine.

¶ Eligible children include those aged ≤18 years who are Medicaid-eligible, uninsured, American Indian or Alaska Native, or insured by health plans that do not fully cover routine immunization (if vaccination is received at a Federally Qualified Health Center or a rural health clinic). https://www.cdc.gov/vaccines/programs/vfc/

** NIS-Child used a landline-only sampling frame during 1995–2010. During 2011–2017, the survey was conducted using a dual-frame design, with both mobile and landline sampling frames included. During 2018, NIS-Child returned to a single-frame design, with all interviews conducted by mobile telephone.

†† Children with at least one vaccination reported by a provider and those who had received no vaccinations were considered to have adequate provider data. “No vaccinations” indicates that the vaccination status is known because the parent or guardian indicated there were no vaccinations and the providers returned no immunization history forms or returned them indicating that no vaccinations had been administered.

§§ The Council of American Survey Research Organizations (CASRO) household response rate is calculated as the product of the resolution rate (percentage of the total telephone numbers called that were classified as nonworking, nonresidential, or residential), screening completion rate (percentage of known households that were successfully screened for the presence of age-eligible children), and the interview completion rate (percentage of households with one or more age-eligible children that completed the household survey). The CASRO household response rate is equivalent to the American Association for Public Opinion Research type 3 response rate (https://www-archive.aapor.org/AAPOR_Main/media/publications/Standard-Definitions20169theditionfinal.pdf). CASRO response rates and the proportions of children with household interviews that had adequate provider data for survey years 2015–2020 are available online. https://www.cdc.gov/vaccines/imz-managers/nis/downloads/NIS-PUF20-DUG.pdf

¶¶ The birth dose of HepB is measured as the proportion of children who received a dose of HepB by age 3 days.

*** Rotavirus is assessed at age 8 months to reflect the maximum age at administration recommended by ACIP.

††† Children waiting 12–18 months to receive the second dose of HepA might receive it during the beginning of the catch-up period, which starts at age 24 months.

§§§ 45 C.F.R. part 46.102(l)(2); 21 C.F.R. part 56; 42 U.S.C. Sect. 241(d); 5 U.S.C. Sect. 552a; 44 U.S.C. Sect. 3501 et seq.

¶¶¶ “Other insurance” includes the Children’s Health Insurance Program, military insurance, coverage via the Indian Health Service, and any other type of health insurance not mentioned elsewhere.

**** MSA status was determined based on household reported city and county of residence and was grouped into three categories: MSA principal city, MSA nonprincipal city, and non-MSA. MSAs and principal cities were as defined by the U.S. Census Bureau (https://www.census.gov/programs-surveys/metro-micro.html). Non-MSA areas include urban populations not located within an MSA and completely rural areas.

††††
https://health.gov/healthypeople/objectives-and-data/browse-objectives/vaccination

§§§§
https://www.cdc.gov/vaccines/imz-managers/nis/downloads/NIS-PUF21-DUG.pdf

¶¶¶¶
https://www.cdc.gov/measles/cases-outbreaks.html
